# High resolution preparation of monocyte-derived macrophages (MDM) protein fractions for clinical proteomics

**DOI:** 10.1186/1477-5956-7-4

**Published:** 2009-02-19

**Authors:** Rita Polati, Annalisa Castagna, Alessandra Bossi, Natascia Campostrini, Federica Zaninotto, Anna Maria Timperio, Lello Zolla, Oliviero Olivieri, Roberto Corrocher, Domenico Girelli

**Affiliations:** 1 University of Verona, Department of Biotechnology, 37134 Verona, Italy; 2 University of Verona, Department of Clinical and Experimental Medicine, Unit of Internal Medicine B, 37134 Verona, Italy; 3 University of Tuscia, Department of Environmental Sciences, 01100 Viterbo, Italy

## Abstract

**Background:**

Macrophages are involved in a number of key physiological processes and complex responses such as inflammatory, immunological, infectious diseases and iron homeostasis. These cells are specialised for iron storage and recycling from senescent erythrocytes so they play a central role in the fine tuning of iron balancing and distribution. The comprehension of the many physiological responses of macrophages implies the study of the related molecular events. To this regard, proteomic analysis, is one of the most powerful tools for the elucidation of the molecular mechanisms, in terms of changes in protein expression levels.

**Results:**

Our aim was to optimize a protocol for protein fractionation and high resolution mapping using human macrophages for clinical studies. We exploited a fractionation protocol based on the neutral detergent Triton X-114. The 2D maps of the fractions obtained showed high resolution and a good level of purity. Western immunoblotting and mass spectrometry (MS/MS analysis) indicated no fraction cross contamination. On 2D-PAGE mini gels (7 × 8 cm) we could count more than five hundred protein spots, substantially increasing the resolution and the number of detectable proteins for the macrophage proteome. The fractions were also evaluated, with preliminary experiments, using Surface Enhanced Laser Desorption Ionization Time of Flight Mass Spectrometry (SELDI-TOF-MS).

**Conclusion:**

This relatively simple method allows deep investigation into macrophages proteomics producing discrete and accurate protein fractions, especially membrane-associated and integral proteins. The adapted protocol seems highly suitable for further studies of clinical proteomics, especially for the elucidation of the molecular mechanisms controlling iron homeostasis in normal and disease conditions.

## Background

Macrophages are involved in a number of key physiological processes and complex responses such as inflammatory, immunological, infectious diseases and iron homeostasis. Iron homeostasis is mainly controlled by the liver-produced hepcidin peptide [1]. This small hormone synchronizes systemic iron fluxes by binding to the iron export channel ferroportin located on the surface of macrophages, hepatocytes and intestinal enterocytes to cause its internalization and proteolysis [2]. Ferroportin, the only known cellular iron exporter, is highly expressed on cells involved in iron export, including the duodenal mucosa, macrophages and cells of the placenta. In macrophages, ferroportin is required for the efficient recycling of iron from ingested erythrocytes [3].

*In vivo*, tissue macrophages are derived from circulating monocytes recruited in the tissues by constitutive or inflammatory signals [4,5]. Primary cultures of monocyte-derived macrophages (MDMs) constitute a good model for studying the biological activities of macrophages, and are excellent candidates for a proteomic approach; in fact they can be easily obtained and cultured within 12 days. During this period they acquire many of the characteristics of *in vivo *activated tissue macrophages, such as CD14 (LPS receptor)-expression [6], and the secretion of proteases involved in remodelling the extracellular matrix [7].

Proteomic analysis is the most powerful method to elucidate the proteic effectors of cellular processes [8-10]. Two-dimensional electrophoresis allows to map protein populations, to identify and underpin proteins whose expression levels correlate with particular responses or with pathological states [11], generating information to designate protein markers specific for the disease. Sometime, the analysis of total cell proteome poses practical challenges, due to its complexity (a thousand of proteins expressed in a cell), to the great dynamic range of protein expression and to the different protein properties (pI, molecular mass, hydrophobicity, post-translational modifications). Suitable strategies to decrease such high complexity are aimed at analysing subsets of the proteome, e.g. by narrowing the pH range used for the first dimension [12], or by the sub-fractionation of proteins into more homogeneous classes [13].

The analysis of single cellular compartments, fractionating the proteins into common localisation categories, e.g. secreted components, membrane, nuclear, organelle's proteins and cytosol, has given important practical advantages and results offer a better insight into the protein expression of each cell fraction considered [14-18]. Sometimes the isolation of proteome sub-sets has been achieved with selective tagging methods for proteins, as in the case of surface proteins, membrane-associated components [19]. Alternatively, sequential extraction methods are used to collect proteins with physico-chemical properties in-common [20].

Aimed at understanding the molecular mechanisms occurring during the physiological responses of macrophages to different stimuli/environment/pathological conditions, the proteome of such cells has been sub-mapped in secretome, cytosol and membrane proteomes [21,22]. Further optimisation of the protein extraction method would results in higher resolution of the 2D maps, with benefit in terms of comparative proteome studies, thus permitting to expand significantly our knowledge on macrophages and on their role in iron dealing. MDMs are a good model for macrophages proteomic studies, being easy to recruit, grow and mimicking well tissues differentiated ones.

Here we report on the effective fractionation of cytosol and membrane proteins of MDMs, by the adaptation of a protocol that uses the neutral detergent Triton X-114, whose peculiarity is the temperature-dependent solubility. The treatment proved to be very effective for fractionating proteins on the basis of their hydropathicity [23]. Membrane, cytosol and secretome proteins have been run on 2D gels. Mini gels allowed to count over 500 protein spots, with very sharply focused spots. MS/MS on sampled spots was used for deciphering the maps, indicating good correlation between the fraction analysed and the protein spot identified in the fraction. In preliminary experiments we also assessed the obtained fraction by SELDI-TOF-MS for hepcidin content, and we could detect a peptide with the same mass as hepcidin only in the cytosolic fraction, as expected.

## Results & Discussion

Primary cultures of human MDMs were prepared by differentiation of monocytes from blood donors according to literature [24]. Optical microscope analysis showed a good differentiation of MDMs in 12 days. The purity of the cultures was evaluated as in ref. [24]; the flow cytometry analysis permitted to assess the purity of the cultures by testing positivity of macrophages to CD14 and CD45 (data not shown). Iron metabolism and macrophages are closely linked; these specialized cells are devoted to iron storage and recycling, expressing crucial proteic effectors on the cell membrane such as ferroportin (the only known iron-exporting-channel) and other soluble iron-related proteins (IronRegulatoryProteins-IRPs, Ferritin, etc.) [24]. While most of the genes and RNAs involved in iron homeostasis have been described, still little is known on the proteins deputed to such function. To unravel protein candidates of clinical interest, proteome analysis appears as the reference technology for such investigations.

Due to the complexity of an entire cell lysate from a proteomic point of view, and willing to gain a wide range of information, we decided to fractionate the sample prior to 2D electrophoresis.

**Figure 1:**
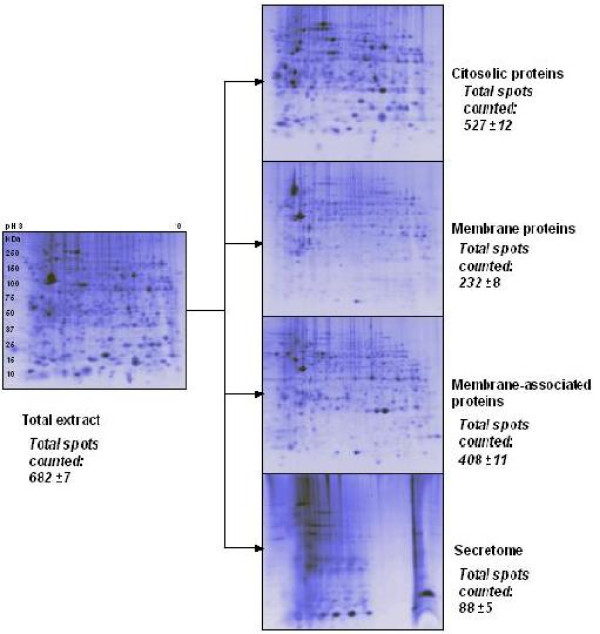
**2D-Maps representing total protein extract from macrophages and protein fractions derived from the proposed protocol**. 2D mini gels of a total protein extract from macrophages cells (panel on the left) and the 4 fractions obtained by applying the extraction protocol with Triton X114 (cytosolic fraction, membrane fraction, membrane associated fraction and secretome fractions, from the top to the bottom). All the 2D-PAGE are run on a 3–10 non linear IPG strip and an equal amount of total protein content was loaded on each gel. For each 2D map the spots count, as obtained with PDQuest software, is indicated.

Protein extraction from MDMs was obtained using a lysis and fractionation protocol which distinguished proteins on the basis of their hydropathicity. This method partially derives from what achieved in [22,23] but was optimised for MDM cell and allowed the sub-fractionation of the total proteome of MDMs into intracellular proteome, membrane proteome, membrane associated proteome and secretome. In Figure 1 2D maps of the 4 fractions are compared with the total lysate normally obtained with a single step sample preparation, clearly showing the increase in resolution and amount of information obtainable. For each fraction the number of protein spots detected is also reported. Being most interested in the membrane fraction, we extracted membrane proteins of macrophages using the neutral detergent Triton X-114. The fractionation protocol exploits the temperature dependent solubility of Triton X-114. Fractionation steps include mixing Triton X-114 with cell lysates at 0°C, where it is freely soluble and where it forms complexes associating hydrophobic proteins, followed by a step-wise change in temperature, reaching Triton X-114 cloud point (23°C), that induce the detergent precipitation, causing the trapping of the protein complexes in an insoluble phase. The treatment proved to be simple and very effective for fractionating membrane and cytosol proteins, permitting to obtain high resolution 2D gels. A fraction of "membrane associated" proteins was also recovered. The use of neutral detergent Triton X-114 for MDM membrane protein extraction was already demonstrated to give better results in comparison to solvent extraction [22], even if it was used only in conjunction with liquid chromatography separations. Here we employed Triton X-114 and analysed extracted proteins by 2D electrophoresis and MS. Upon lysis of human MDMs, the intracellular fraction was recovered by precipitation of the supernatant. The pellet of the lysis, containing the membranes was treated with the neutral detergent Triton X-114 for protein extraction. The protocol was adjusted for MDMs: the re-iteration in sequence of the mixing steps with the detergent and incubation steps ameliorated the protein-detergent complexation.

**Figure 2:**
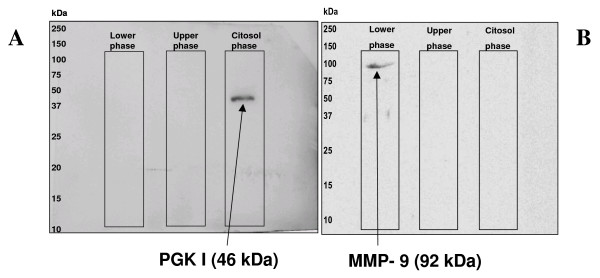
**Western blot analysis for PGK-1 presence in the obtained fractions**. Western blot analyses for the cytosolic enzyme phosphoglycerate kinase (PGK I), typical of the glycolysis, panel A, and for the membrane bound protein Metalloproteinase 9 (MMP9), panel B. The three fractions were run together on the same SDS-PAGE loading an equal amount of total protein. The same PVDF membrane was used. Film image with the relative protein bands only in the lane corresponding to the cytosolic compartment and to the membrane fraction are reported. Western blot images were captured by GS710 densitometer (Bio-Rad) and analyzed by QuantityOne software.

Fractions were run on 2D-PAGE (Minigel of 8 cm length) and maps were analysed, after staining with Sypro Ruby, by PDQuest software. After images digitalization it was possible to highlight the presence of an elevated number of spots in all the maps representative of the fractions (i.e. 527 ± 12, 232 ± 8, 408 ± 11 in the intracellular, membrane and membrane associated fraction, respectively). These spots number accounts for a good quality and high resolution protein profiles. The quality of the fractions recovered (intracellular and membrane) was assessed by SDS-PAGE electrophoresis followed by MS-MS or Western blotting. Intracellular fraction was assayed for the presence of the cytosolic enzyme phosphoglycerate kinase (PGK I), a protein typical of the glycolytic pathway and for the absence of membrane protein contamination, (Figure 2, panels A-B), by incubating the same Polyvinylidene Fluoride (PVDF) membrane with an antibody against the matrix metallopeptidase 9 (MMP-9).

The three main fraction were run on the same SDS-PAGE and equal amount of total protein were loaded in each lane. Only the lane referring to the cytosolic fraction showed a distinct band when developed with the anti-PGK1 antibody, being clear the presence of the enzyme only in there and thus indicating no major contamination between fractions. In addition, only the lane referring to the membrane fraction showed a distinct band when developed with the anti MMP-9 antibody, indicating no contamination of the cytosolic fraction by membrane proteins.

**Figure 3:**
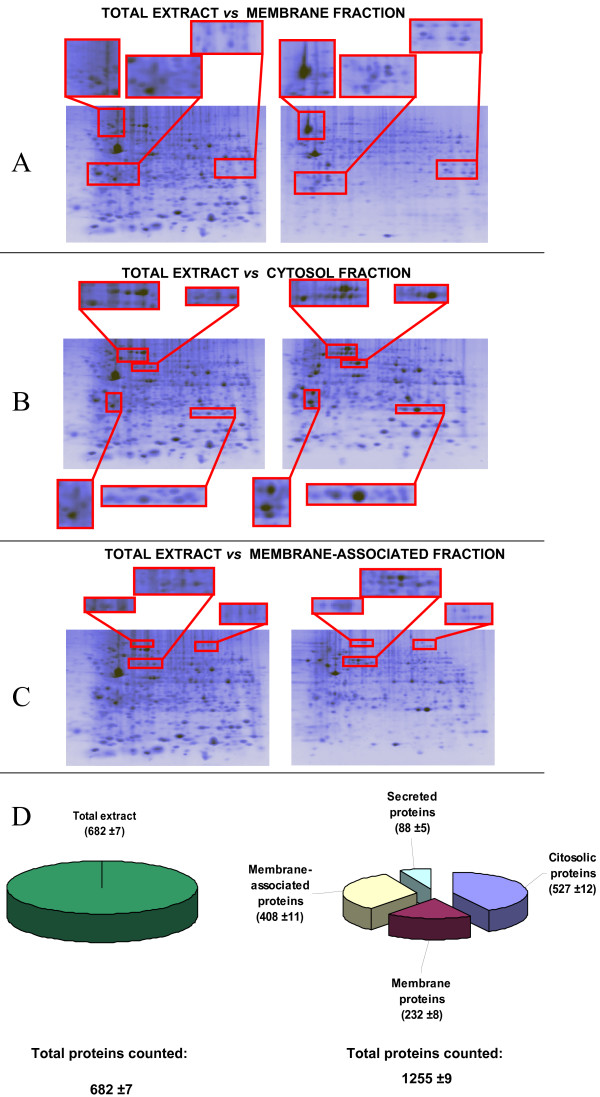
**Comparison between total extract 2D map and fractions 2D maps**. 2D gels of total extract and fractions with enlargements of specific zones and a diagram reassuming the numerical data obtained. A) total extract versus membrane fraction. B) total extract versus cytosolic fraction. C) total extract versus membrane associated fraction. D) Pie chart representing the Spots counts relative to the total extract (left panel) and the summary of the 4 fractions obtained with the proposed protocol.

**Figure 4:**
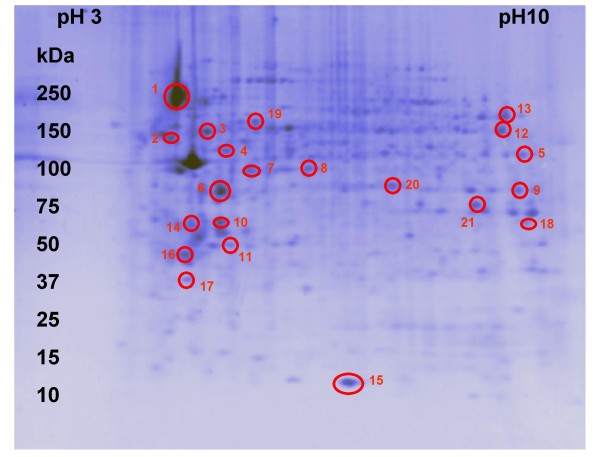
**2D map of the membrane fraction used for spot excision and ID**. 2D gel of the membrane fraction showing the cut spots as red circles numbered from 1 to 21.

Figure 3 shows some comparisons between selected zones of the total extract and the corresponding zone on 2D gels of fractions, highlighting the different resolution achieved when proteins where fractionated prior to the 2D run. In analogy with membrane proteome literature [25], the recovery of integral and membrane associated proteins was successfully obtained with the use of detergents, because their lipophilic character mimics the native lipid-membrane environment. A quantitative evaluation of the 2D maps was performed by comparing the results of spots image analysis of the membrane fraction with those of the total extract. It was evident that, in the selected zones (Figure 3), the increase in spot density resulted 2.1 fold, thus indicating the substantial improvement in density gained with the fractionation. The membrane fraction was also subjected to qualitative studies. MS analysis of a sample of 21 spots from the membrane fraction gave the identifications shown in Table 1 (the cut spots are indicated with circles in the map in Figure 4). Among these proteins, 10 are integral membrane proteins and the remaining proteins are all associated to the plasma membrane or to membrane proteins, being 90% the total recovery percentage. These results support the Triton X114 extraction method for MDMs membrane protein studies. Figure 3D reports the number of protein counted in each fraction and the count of the map of the total extract. The number of proteins doubles in case of fractionation, also indicating the great advantage of the chosen protocol. The fractionation of the samples enabled to increase the amount of information achievable on the protein effectors in macrophages. All the four compartments analysed can be further studied and evaluated in terms of specific enzymes or antigens.

**Table 1:** membrane fraction identified proteins

**SSP**	**Mw, kDa theor./exp**.	**pI predict./exp**.	**No. of peptides identified**	**Mascot Score**	**NCBI Accession Number**	**Protein ID**
**5**	51372	8.88	11	592	gi|872121	**isocitrate dehydrogenase (NADP+)** **[Homo sapiens]**

	38780	7.57	21	1301	gi|18645167	**Annexin A2** **[Homo sapiens]**

**21**	38639	6.32	3	162	gi|190200	**Porin** **[Homo sapiens]**

	42128	5.22	4	198	gi|28336	**mutant beta-actin (beta'-actin)** **[Homo sapiens]**

**17**	18537	5.21	2	70	gi|5453559	**ATP synthase, H+ transporting, mitochondrial F0 complex, subunit d isoform a** **[Homo sapiens]**

	58411	7.58	17	1101	gi|35505	**pyruvate kinase** **[Homo sapiens]**

**13**	59828	9.16	2	113	gi|4757810	**ATP synthase, H+ transporting, mitochondrial F1 complex, alpha subunit precursor** **[Homo sapiens]**

**1**	53738	5.03	36	1940	gi|340219	**Vimentin** **[Homo sapiens]**

**14**	42080	5.37	6	317	gi|62897625	**beta actin variant** **[Homo sapiens]**

**15**	12905	5.77	3	70	gi|34616	**beta-2 microglobulin** **[Homo sapiens]**

**12**	59785	9.07	20	1136	gi|127798841	**ATP synthase, H+ transporting, mitochondrial F1 complex, alpha subunit 1, cardiac muscle** **[Homo sapiens]**

**10**	29843	5.57	9	485	gi|4505773	**prohibitin** **[Homo sapiens]**

**3**	50810	5.02	25	651	gi|37492	**alpha-tubulin** **[Homo sapiens]**

**18**	30737	8.63	7	420	gi|238427	**Porin 31HM [human, skeletal muscle membranes, Peptide, 282 aa]** **[Homo sapiens]**

**11**	30337	6.99	3	178	gi|4758788	**NADH dehydrogenase (ubiquinone) Fe-S protein 3, 30 kDa (NADH-coenzyme Q reductase)** **[Homo sapiens]**

**20**	49851	7.70	20	710	gi|704416	**elongation factor Tu** **[Homo sapiens]**

**16**	42052	5.29	5	131	gi|4501885	**beta actin** **[Homo sapiens]**

**7**	42080	5.37	8	398	gi|62897625	**beta actin variant** **[Homo sapiens]**

**2**	48083	4.95	17	1052	gi|89574029	**mitochondrial ATP synthase, H+ transporting F1 complex beta subunit** **[Homo sapiens]**

**8**	53559	5.93	4	189	gi|32709	**IFP53** **[Homo sapiens]**

**6**	42080	5.37	8	371	gi|62897625	**beta actin variant** **[Homo sapiens]**

**4**	53297	5.94	6	241	gi|46593007	**ubiquinol-cytochrome c reductase core protein I** **[Homo sapiens]**

**19**	53809	6.03	19	1278	gi|40889610	**Chain A, Crystal Structure Of Human Tryptophanyl-Trna Synthetase** **[Homo sapiens]**

Identified proteins from membrane fraction. The corresponding standard spot number (SSP) and the parameters of identifications are also indicated.

Being interested in iron metabolism we also evaluated the fractions in term of hepcidin content (see the additional files 1 and 2 for SELDI-TOF-MS method details and figure). Hepcidin is the master regulator of iron homeostasis and acts by tuning iron influx into plasma from tissues dedicated to iron storage or transport. In particular macrophages recycle iron from senescent entherocytes. Our interest was to monitor hepcidin presence in the fraction in order to study its behavior in cultured macrophages. In preliminary experiments, a peptide matching the mass of hepcidin was detectable only in the cytosol but not in the other compartments (see additional file 2).

Notwithstanding recent progress, much work remains to be done in defining the role of hepcidin in both healthy and diseased states. However, to date, few investigative tools are available [26-29]. By means of SELDI-TOF-MS technology, we and others were successful to detect hepcidin and its isoforms in urine and serum [1,30,31]. Our preliminary results appear to confirm the presence of hepcidin in macrophages also as protein, extending the data reported by Theurl and colleagues about hepcidin mRNA in monocyte/macrophages [32]. The presence of a peptide of the same mass as hepcidin in the cytosolic fraction is in agreement with the known cycle of hepcidin from liver to cells, by means of binding to ferroportin and internalization [1,2,33]. We are going to validate this approach investigating other MDMs under different conditions. Further experiments are needed for a better understanding of the peptide behavior regarding its binding to membrane proteins. The mutual interaction of hepcidin with ferroportin is essential for the understanding of iron homeostasis in the cells [34] and the study of MDMs from patients and/or animal models of ferroportin disease [35] could give new insights into this field.

## Conclusion

The purpose of this work was to find a feasible method for the study of cytosolic, integral membrane, membrane associated and secreted proteins in comparative proteomics experiments of clinically relevant samples. This technique, based on Triton X-114, allowed us to obtain high resolution 2D maps for all the fractions. The fractionation and extraction method gave as an improvement in spots number detectable and amount of information achievable. In particular the results obtained mapping membrane proteins are remarkable: the maps show high quality spots and no streaks. In fact membrane proteins, due to their hydrophobicity, usually focus poorly using the conventional isoelectrofocusing (IEF) procedure, often leading to horizontal streaks. Mapping separately the protein population of macrophages, in healthy and disease conditions, would allow a deeper understanding of Hereditary Hemochromatosis and iron related disorders.

## Methods

### Monocyte Derived Macrophages Coltures

Peripheral blood mononuclear cells were isolated from healthy human blood donors attending to the Transfusion Service, University Hospital of Verona.

Primary cultures of human MDMs were prepared as described by Pinet [24] with minor changes. Monocytes were left to differentiate in a RPMI medium, containing 2 mM streptomycin, 2 mM Gln, and 10% FCS. Monocytes purity was tested by flow cytometer, according to ref [24] quality criteria.

### Cell lysis

After the differentiation, cells were lysed as in ref [22] with some modifications. Lysis solution was 10 mM HEPES, 10 mM KCl, protease inhibitor (Mini-Complete Roche). Cell were washed 4–5 times with 10 mL DPBS at room temperature. The washes were collected and pooled for secretome analysis. Three mL of cold lysis buffer were added and incubate 10 min. Cells were scraped from the surface and centrifuged 25 min at 16000 g. Cytoplasm was recovered as supernatant, membranes as pellet.

### Extraction of secreted proteins

The culture media were pooled and the proteins precipitated for 1 h at 0°C by adding 15% TCA. The protein pellets were collected by centrifuging at 13000 g for 10 minutes at 4°C and washed two times with 1 mL of cold acetone. The pellets were then resuspended in buffer containing 2 M tiourea, 7 M urea, 3% CHAPS, 20 mM Tris, and protein concentration was determined by Bradford assay using BSA as standard.

### Extraction of intracellular (cytosol) proteins

The supernatant was precipitated overnight at -20°C with acetone: methanol (8:1 vol/vol), then centrifuged 20 min at 18300 g; the pellet was recovered, let dry and re-suspended in 7 M urea, 2 M thiourea, 3% CHAPS, 20 mM Tris, 1% ampholytes and centrifuged 40 min at 18300 g to precipitate DNA contaminants (dark pellet on the bottom of the eppendorf).

### Extraction of membrane proteins

The extraction buffer is prepared with 2% Triton X-114 in TBS (150 mM NaCl, 10 mM Tris-HCl pH 7.6). The extraction is conducted on ice. The membrane pellet is re-suspended in 100 μl MilliQ water and added of 500 μl extraction buffer, then 1) homogenised with a small syringe, 2) let stand in ice for 1 min, 3) vortex for 1 min, 4) let stand in ice for 1 min. The four steps are repeated five times and then the sample is kept on ice for 10 min, vortexed, and finally put 1 hour at 37°C. The sample is then centrifuged for 5 min at 16000 g at room temperature. The lower phase contains Triton X-114 with the membrane proteins, the upper phase contains the aqueous phase and proteins. The two phases are collected and each one is precipitated overnight at -20°C with acetone: methanol (8:1 vol/vol), then centrifuged 20 min at 18300 g. Each pellet was recovered, let dry and re-suspended in 7 M urea, 2 M thiourea, 3% CHAPS, 20 mM Tris.

### Extraction of membrane-associated proteins

The upper phase, expected to be enriched in hydrophilic proteins, collected during the extraction of membrane proteins, was precipitated overnight with cold acetone:methanol (8:1 vol/vol) at -20°C. The protein pellets were recovered by centrifugation at 18300 g for 20 minutes at 4°C. The pellets were then resuspended in buffer containing 2 M thiourea, 7 M urea, 3% CHAPS, 20 mM Tris, and protein concentration was determined by Bradford assay.

### Control of fraction purity by western immunoblotting

Protein fractions were separated by SDS-PAGE and immunodetected with antibody specific for a cytosolic protein (PGK I) and a membrane bound protein (MMP9) by Western blot to verify the efficiency of separation protocol. Protein extracts were diluited 1:1 with Laemmli's sample buffer (62.5 mM Tris-HCl, pH 6.8, 25% glycerol, 2% SDS, 0.01% bromophenol blue, 5% β-mercaptoethanol), boiled for 3 min and separated by SDS-PAGE on 12% T acrylamide gels in Tris/glycine/SDS buffer. Proteins were then electroblotted onto PVDF membranes (Biorad) at 60 V for 2 h at 4°C. Non specific sites were blocked by incubating with 3% non-fat dried milk and 0.05% Tween-20 (Sigma-Aldrich) in Tris-buffered saline (TBS-T) for 1 h at 37°C. Membranes were incubated overnight at room temperature with the primary antibody for PGK I (Sigma-Aldrich) diluited 1:500, in 3% non-fat dried milk and 0.05% Tween-20 (Sigma-Aldrich) in TBS and with the primary antibody for MMP-9 (Sigma-Aldrich) diluted 1:1000 in 3% non-fat dried milk and 0.05% Tween-20 (Sigma-Aldrich) in TBS. Membranes were washed four times for 15 min with TBS-T and then were incubated for 1 h at room temperature with the appropriate horseradish peroxidase-conjugated secondary antibody: ECL anti-goat IgG horseradish peroxidase-linked (Sigma-Aldrich) at 1:20000 diluition for PGK I and ECL anti-rabbit IgG horseradish peroxidase-linked (Sigma-Aldrich) at 1:5000 diluition for MMP-9. Membranes were washed three times for 15 min with TBS-T and once for 15 min with TBS. Finally the immunocomplexes were detected by chemiluminescence (ECL, GE Healthcare,) on X-ray X-Omat AR (Kodak, Rochester, NY, USA) films. The Western-blot image was obtained by scanning films using Quantity One software Version 4.4 (Biorad).

### 2D electrophoresis

Proteins samples (100 μg for intracellular proteins, 100 μg for membrane proteins, 100 μg for membrane-associated proteins and 100 μg for secreted proteins) were mixed with solubilization buffer (2 M thiourea, 7 M urea, 3% CHAPS, 20 mM Tris) to obtain a final volume of 150 μl. Each sample was reduced and alkylated with 5 mM tributylphosphine and 10 mM acrylamide. The mixture was then applied to the dry gel strip (IPG 70 mm, pH 3–10 non linear gradient) for reswelling. Focusing was performed at 300 V for 2 h, 400 V for 1 h, 1000 V for 6 h, 2000 V for 2 h, 3500 V for 5 h, 5000 V until the complete focalization (25000 Vxh). The current was limited to 50 μA *per *strip, and the temperature was kept at 20°C for all IEF steps. For SDS-PAGE, the IPG strips were incubated in equilibration buffer (6 M urea, 2% SDS, 20% glycerol, 0.375 M Tris-HCl pH 8.8) for 26 minutes and then transferred to the second dimension onto 10%–20% T gradient acrylamide gels. The gels were run 5 mA *per *gel for 1 h, 10 mA *per *gel for 1 h and 20 mA *per *gel until the bromophenol blue front had reached the bottom of the gel. The 2-DE gels were stained in Sypro Ruby: the proteins were first fixed in a solution of 7% acetic acid and 10% methanol for 1 h, then incubated in Sypro Ruby overnight and finally destained in 7% acetic acid and 10% methanol for 2 h. Sypro Ruby stained 2-DE gels were digitized using VersaDoc (BioRad, Hercules, CA) and bioinformatic analysis was performed with PDQuest 7.3.0 (BioRad).

### Mass spectrometry analysis

#### In-Gel-Digestion

Protein spots were carefully cut out from Sypro Ruby stained gels and subjected to in-gel trypsin digestion according to Shevchenko and colleagues with minor modifications [36]. The gel pieces were swollen in a digestion buffer containing 50 mM NH_4_HCO_3 _and 12.5 ng/μL of trypsin (modified porcine trypsin, sequencing grade, Promega, Madison, WI) in an ice bath. After 30 min, the supernatant was removed and discarded, 20 μL of 50 mM NH_4_HCO_3 _was added to the gel pieces, and digestion was allowed to proceed at 37°C overnight. The supernatant containing tryptic peptides was dried by vacuum centrifugation. Prior to mass spectrometric analysis, the peptide mixtures were redissolved in 10 μL of 5% Formic Acid.

#### Protein Identification by nano-HPLC-MS/MS

Peptide mixtures were separated using a nanoflow-HPLC system (Ultimate; Switchos; Famos; LC Packings, Amsterdam, The Netherlands). A sample volume of 10 μL was loaded by the autosampler onto a homemade 2 cm fused silica precolumn (75 μm i.d.; 375 μm o.d.; Reprosil C18-AQ, 3 μm (Ammerbuch-Entringen, DE)) at a flow rate of 2 μL/min. Sequential elution of peptides was accomplished using a flow rate of 200 nL/min and a linear gradient from solution A (2% acetonitrile and 0.1% formic acid) to 50% of solution B (98% acetonitrile and 0.1% formic acid) in 40 min over the precolumn in-line with a homemade 10–15 cm resolving column (75 μm i.d.; 375 μm o.d.; Reprosil C18-AQ, 3 μm (Ammerbuch-Entringen, Germany)).

Peptides were eluted directly into a High Capacity ion Trap (model HCTplus, Bruker-Daltonik, Germany). Capillary voltage was 1.5–2 kV and a dry gas flow rate of 10 L/min was used with a temperature of 230°C. The scan range used was from 300 to 1800 *m*/*z*. Protein identification was performed by searching in the National Center for Biotechnology Information nonredundant database (NCBInr) using the Mascot program . The following parameters were adopted for database searches: complete carbamidomethylation of cysteines and partial oxidation of methionines, peptide mass tolerance ± 1.2 Da, fragment mass tolerance ± 0.9 Da, missed cleavages 2. For positive identification, the score of the result of (-10 Log(*P*)) had to be over the significance threshold level (*P *< 0.05).

Even though high MASCOT scores are obtained with values greater than 60, when proteins were identified with only one peptide, a combination of automated database search and manual interpretation of peptide fragmentation spectra was used to validate protein assignments. In this manual verification, the mass error, the presence of fragment ion series, and the expected prevalence of C-terminus containing (Y-type ions) in the high mass range were all taken into account. Moreover, replicate measurements have confirmed the identity of these protein hits.

## Competing interests

The authors declare that they have no competing interests.

## Authors' contributions

RP made substantial contributions to proteomic data acquisition and analysis, and co-wrote the manuscript. AC made substantial contributions to conception and design, and co-wrote the paper. AB contributed to conception and design, and was involved in manuscript drafting and data interpretation. NC was involved in collecting samples for this study and SELDI analysis and interpretation. FZ contributed to experimental analysis and set up. AMT and LZ performed MS-MS analysis and data interpretation. OO contributed to conception and design, data analysis and interpretation. RC participated in the design, coordination of the study and manuscript drafting. DG made substantial contributions to conception and design and revised critically the manuscript for important intellectual content. All authors have read and approved the final manuscript.

## Supplementary Material

Additional file 1 **Method for seldi-tof analysis.** it contains a description of the methodology we used for performing the SELDI-TOF ANALYSIS Click here for file

Additional file 2 **Figure**1-**SELDI analysis of the fractions.** It contains figure 1 with the SELDI spectra of the fractions investigated and a spectrum with the synthetic standard spiked in the cytosolic fraction along with the figure caption. Click here for file
