# Supplementary material for: High resolution preparation of monocyte-derived macrophages (MDM) protein fractions for clinical proteomics
Source: Proteome Sci. 2009 Feb 19;7:4. doi: 10.1186/1477-5956-7-4 (PMC2649903; doi:10.1186/1477-5956-7-4)
Supplement: Additional file 1 — Method for seldi-tof analysis. it contains a description of the methodology we used for performing the SELDI-TOF ANALYSIS [file 1477-5956-7-4-S1.pdf]

METHOD FOR SELDI-TOF- ANALYSIS

Hepcidin-25 measurements in the MDM fractions were performed by SELDI-TOF-MS, using a Ciphergen Protein Biosystem IIc mass spectrometer (model PBSIIc, Ciphergen Biosystems, Freemont, CA) and IMAC 30 ProteinChip arrays (Bio-Rad Laboratories, Hercules, CA), according to the manufacturer's instructions with some modifications. A similar protocol was used for semi-quantitative detection of urinary hepcidin [31]. Finally, 1 μl of a saturated solution of sinapinic acid in 0.5% (vol/vol) trifluoroacetic acid and 50% (vol/vol) acetonitrile, used as energy-absorbing matrix (EAM), was applied to each spot surface, allowed to air-dry, and reapplied. Mass-to-charge (*m*/*z*) spectra were generated using the PBSIIc TOF mass spectrometer at laser intensity 180, detector sensitivity 9; high mass limit 50 kDa; and optimization interval 1500 to 10000 Da. Synthetic 25-hepcidin (Peptides International, Louisville, KY) was used for external mass calibration and, spiked into the cytosolic fraction sample, for peak confirmation. Peak annotation was performed with ProteinChip Software after baseline subtraction
