# Supplementary material for: High resolution preparation of monocyte-derived macrophages (MDM) protein fractions for clinical proteomics
Source: Proteome Sci. 2009 Feb 19;7:4. doi: 10.1186/1477-5956-7-4 (PMC2649903; doi:10.1186/1477-5956-7-4)
Supplement: Additional file 2 — Figure1-SELDI analysis of the fractions. It contains figure 1 with the SELDI spectra of the fractions investigated and a spectrum with the synthetic standard spiked in the cytosolic fraction along with the figure caption. [file 1477-5956-7-4-S2.pdf]

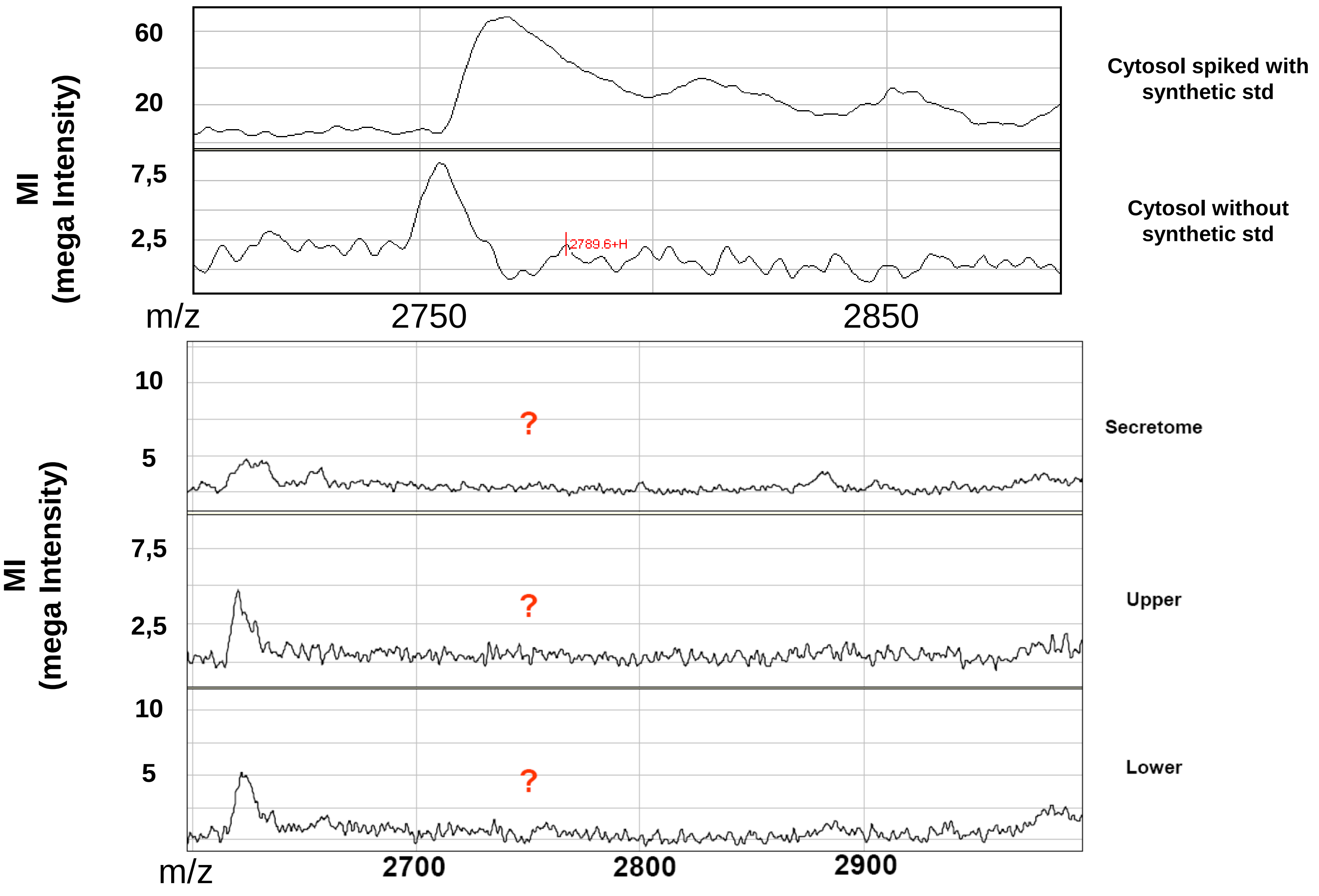

Figure 1 – SELDI analysis of the fractions.

SELDI-TOF spectra representing the four fractions analysed on IMAC 30 chip arrays. The peak of a peptide matching the mass of hepcidin 25 is shown when present. A spectrum containing the hepc 25 synthetic standard spiked into the cytosolic fraction is also shown.
